# EPAS1 amplifies asthma pathogenesis through JAK2/STAT3-mediated ferroptosis and inflammation

**DOI:** 10.17305/bb.2025.11334

**Published:** 2025-03-27

**Authors:** Lili Liu, Cheng Yang, Yan Li, Hao Zhou, Mei Shi, Tiantian Shi, Weibing Shi

**Affiliations:** 1 The First Affiliated Hospital of Anhui University of Chinese Medicine, Hefei, Anhui, China; 2 The Second Affiliated Hospital of Anhui University of Chinese Medicine, Hefei, Anhui, China; 3 Anhui University of Chinese Medicine, Hefei, Anhui, China

**Keywords:** Asthma, ferroptosis, JAK2/STAT3, endothelial PAS domain protein 1, EPAS1, inflammation

## Abstract

Asthma is a chronic respiratory disorder marked by airway hyperresponsiveness and inflammation, yet the specific molecular mechanisms driving these processes remain only partially understood. This study aims to better understand how the JAK2/STAT3/EPAS1 axis regulates inflammation and ferroptosis in asthma. Asthma-related datasets were retrieved from the Gene Expression Omnibus (GEO) database, and differentially expressed genes (DEGs) were identified. Weighted Gene Co-expression Network Analysis (WGCNA) was used to detect gene modules associated with asthma. A protein-protein interaction (PPI) network was then constructed by intersecting WGCNA-derived genes with ferroptosis-related genes to identify key hub genes. The diagnostic value of these ferroptosis-associated genes was evaluated using Receiver Operating Characteristic (ROC) curve analysis. Additionally, immune cell infiltration in asthma patients was analyzed using the Immune Cell AI database in relation to ferroptosis-related genes. Functional experiments at the cellular level were conducted to assess the effects of key genes on cell viability, inflammation, and ferroptosis. Bioinformatics analysis identified 1,698 DEGs linked to asthma. Five hub genes with clinical diagnostic value— Endothelial PAS Domain Protein 1 (EPAS1), STAT3, G6PD, CYBB, and CBS—were identified. Immune analysis revealed that EPAS1 is closely associated with immune cell infiltration in asthma. Functional experiments further demonstrated that the JAK2/STAT3 axis promotes ferroptosis and inflammatory responses by upregulating EPAS1 expression. Notably, these findings highlight the JAK2/STAT3/EPAS1 axis as a potential therapeutic target for asthma, offering new insights into its molecular mechanisms and identifying novel biomarkers for diagnosis and treatment.

## Introduction

Asthma is a common respiratory condition characterized by fluctuating airway obstruction, airway hyperreactivity, and persistent airway inflammation [1]. Clinically, it manifests as wheezing, coughing, shortness of breath, and chest tightness [2]. The global incidence of asthma is rising annually, creating a significant public health burden [3]. The pathophysiology of asthma is complex, involving genetic predisposition, environmental factors, and immune system dysfunction [4]. Asthma is typically classified into different subtypes based on the predominant inflammatory cell type, including eosinophilic, neutrophilic, and mixed asthma [5]. Eosinophilic asthma, a key subtype, is primarily driven by eosinophil activation and is often associated with more severe disease and a poorer prognosis [6]. The biological mechanisms underlying eosinophilic asthma involve the release of cytokines such as interleukin-4 (IL-4), interleukin-5 (IL-5), and interleukin-13 (IL-13), which promote eosinophil recruitment, activation, and survival in the airways [7]. These cytokines, along with other mediators, contribute to airway inflammation, mucus production, and structural remodeling. Recent studies have identified novel asthma biomarkers, including fractional exhaled nitric oxide (FeNO), which reflects eosinophilic inflammation, and epithelial barrier proteins, such as tight junction proteins, which play a role in airway epithelial integrity disruption [8]. Current asthma treatments primarily include pharmacological interventions and lifestyle modifications, with inhaled corticosteroids and bronchodilators being the most common medications [9]. In addition, biologic therapies have emerged as an important option, particularly for severe or difficult-to-treat cases. Biologic drugs, such as mepolizumab, benralizumab, omalizumab, reslizumab, and, more recently, tezepelumab, have demonstrated efficacy in reducing exacerbations, improving lung function, and decreasing the need for oral corticosteroids [10]. These biologics work by targeting key inflammatory mediators, such as IL-5 and immunoglobulin E (IgE), offering a more personalized approach to asthma management. Ferroptosis, a newly identified form of programmed cell death, is characterized by excessive intracellular iron accumulation and lipid peroxidation [11]. Unlike traditional apoptosis or necrosis, ferroptosis is driven by abnormal iron deposition and oxidative stress [11]. Recent research has highlighted the role of ferroptosis in various diseases, with a focus on mechanisms, such as iron homeostasis imbalance, lipid peroxidation, and antioxidant system disruption [12]. Significant progress has been made in understanding ferroptosis in respiratory diseases. In chronic obstructive pulmonary disease (COPD), unstable iron accumulation and increased lipid peroxidation in lung epithelial cells—particularly during cigarette smoke exposure—have been linked to non-apoptotic cell death [12]. In idiopathic pulmonary fibrosis, the compound fraxetin has been shown to slow disease progression by inhibiting NCOA4-mediated epithelial cell ferroptosis and reducing inflammatory cytokine release [13]. In asthma, ferroptosis has also been implicated in airway remodeling and inflammatory responses [14]. Elevated iron levels in human airway smooth muscle cells and fibroblasts can trigger proinflammatory cytokine production and extracellular matrix remodeling. In vivo studies suggest that excess iron contributes to key asthma features, such as airway hyperreactivity, fibrosis, and type 2 (T2) inflammation [15]. Additionally, research indicates that the ferroptosis inhibitor ferrostatin-1 may have therapeutic potential in asthma [16]. Understanding the role of ferroptosis in asthma offers valuable insights into its underlying mechanisms. Targeting ferroptosis pathways, particularly in combination with biologic therapies, may provide novel therapeutic strategies for asthma management.

The Janus kinase 2 (JAK2)/signal transducer and activator of transcription 3 (STAT3) pathway is a critical signaling cascade involved in various biological processes (BPs), including immune response, inflammation, and cell survival [17]. JAK2, a tyrosine kinase, is activated by cytokines and growth factors, leading to the phosphorylation and activation of the transcription factor STAT3. Recent studies have highlighted STAT3 as a key regulator of ferroptosis in gastric cancer, where it inhibits tumor growth and mitigates chemotherapy resistance [18]. Endothelial PAS domain-containing protein 1 (EPAS1), primarily responsible for regulating cellular responses to hypoxia, plays a crucial role in erythropoiesis, angiogenesis, and metabolism [19]. Research has also identified EPAS1 as a hypoxia- and ferroptosis-related gene, particularly in diseases characterized by oxidative stress and dysregulated iron metabolism [20]. By analyzing asthma transcriptome data, this study aims to identify key ferroptosis-associated genes and their enriched pathways. Furthermore, it seeks to investigate the regulatory role of the JAK2/STAT3/EPAS1 axis in ferroptosis in asthma. We anticipate that this research will enhance our understanding of asthma-related airway epithelial damage, provide novel therapeutic targets, and facilitate the development of relevant pharmaceutical interventions.

## Materials and methods

### Data acquisition and differentially expressed gene (DEG) screening

We first analyzed genes associated with asthma. Gene expression data for GSE134544 was obtained from the Gene Expression Omnibus (GEO) database (https://www.ncbi.nlm.nih.gov/geo/), which includes profiles from 20 control samples and 41 asthma samples. DEGs were identified using the GEO2R tool. Genes were considered upregulated if they had a fold change (FC) greater than 1.3 and a *P* value less than 0.05. Conversely, genes with an FC lower than 0.77 and a *P* value less than 0.05 were classified as downregulated.

### Enrichment analysis

To investigate the potential biological functions of the genes, we conducted an enrichment analysis. This analysis included three Gene Ontology (GO) domains: BP, Cellular component (CC), and Molecular function (MF). Additionally, pathway datasets related to diseases, drugs, chemicals, and BPs were retrieved from the Kyoto Encyclopedia of Genes and Genomes (KEGG) database. Enrichment analysis was performed using the clusterProfiler R package (v4.8.1) to identify relevant biological functions and associated pathways. The results of the GO and KEGG enrichment analyses were visualized using the online platform Bioinformatics.

### Weighted gene co-expression network analysis (WGCNA)

To identify key modules related to asthma, we performed WGCNA using the R package WGCNA (v1.71). First, we preprocessed the gene expression data by removing low-expression genes and samples with excessive missing values, followed by normalization to ensure data suitability. Next, we constructed a weighted adjacency matrix by calculating the Pearson correlation between gene pairs and raising the correlation coefficients to a soft-thresholding power (β) to achieve a scale-free network topology. The resulting adjacency matrix was then converted into a Topological Overlap Matrix (TOM). Modules were identified through hierarchical clustering, and feature genes were computed. With a merge cut height of 0.25, the soft-thresholding power was set to *β* ═ 12. To determine the association between phenotypes (asthma vs. control samples) and each module, we performed Pearson correlation analysis. This allowed us to identify modules significantly associated with asthma, and the genes within these modules were considered asthma-related module genes.

### Extraction of ferroptosis-related genes

To analyze the association between ferroptosis and asthma, we obtained ferroptosis-related genes from the FerrDb V2 database. FerrDb V2 (http://www.zhounan.org/ferrdb/current/) is a manually curated resource that tracks ferroptosis-related disorders, biomarkers, and regulators [21, 22]. Genes from this database were extracted and compared with asthma-related module genes identified through WGCNA. A Venn diagram (http://bioinformatics.psb.ugent.be/webtools/Venn/) was used to visualize the overlap between these gene sets.

### Interaction network construction and hub gene identification

To analyze gene connections, we constructed a protein–protein interaction (PPI) network. The Search Tool for the Retrieval of Interacting Genes (STRING, https://string-db.org/) was used to investigate PPI networks. Using Cytoscape V3.9.0 (https://cytoscape.org/), we selected PPI networks with a confidence score higher than 0.40 and enhanced their visual presentation. Hub genes were identified using CytoHubba’s Degree calculation, with the top 10 nodes considered hub genes. Additionally, we used GeneMANIA (http://www.genemania.org), an open-source tool, to create and visualize interactive functional association networks, illustrating gene connections.

### Single-gene gene set enrichment analysis (GSEA)

Single-gene GSEA analysis was conducted to identify the potential biological functions of key genes. The correlation coefficient between the target gene expression and total gene expression, which served as the ranking criterion for GSEA, was calculated before running the analysis. The KEGG gene sets were used as the reference. GSEA was performed using the R package clusterProfiler, with a significance threshold of *P* < 0.05.

### Immune infiltration correlation analysis

ImmuneCellAI (https://guolab.wchscu.cn/ImmuCellAI/#!/) is a tool used to estimate the abundance of 24 different immune cell types based on gene expression datasets, including RNA-Seq and microarray data. These 24 immune cells comprise six general immune cell types and 18 T cell subtypes. Using the ImmuneCellAI algorithm, we analyzed the infiltration levels of these immune cells in both control and asthma samples. Pearson correlation coefficients were then used to assess the relationships between immune cell infiltration and ferroptosis-related genes, as well as the correlations among different immune cell types.

### Cell culture and treatment

To investigate the role of key genes, we constructed cellular models of asthma. Human bronchial epithelial cells (16HBE, YS003C, Yaji Bio, China) were cultured in Dulbecco’s Modified Eagle Medium (Gibco, USA) supplemented with 10% fetal bovine serum (FCS500, Excell Bio, China) and maintained in a 37 ^∘^C incubator with 5% CO_2_. Two cell groups were established: interleukin-13 (IL-13) and control (Ctrl). To induce the asthma model, the IL-13 group was treated with 10 ng/mL of IL-13 (C01M, Nearshore Protein, China) for 24 h [23].

### Cell transfection

We investigated the roles of STAT3 and EPAS1 in asthma by overexpressing or knocking down these genes. Control small interfering RNA (si-NC) and STAT3-targeting siRNA (si-STAT3) were designed and provided by GenePharma (China). Overexpression plasmids for STAT3 (OE-STAT3), endothelial PAS domain protein 1 (EPAS1; OE-EPAS1), and a control plasmid (OE-NC) were supplied by Wuhan Miaoling Biotechnology Co., Ltd. For the experiment, 16HBE cells were divided into eight groups: OE-NC, OE-STAT3, si-NC, si-STAT3, si-NC+OE-NC, si-STAT3+OE-NC, si-NC+OE-EPAS1, and si-STAT3+OE-EPAS1. Transfections were performed using Lipofectamine 2000 (12566014; Thermo Fisher Scientific, USA) according to the manufacturer’s instructions. After 48 h of transfection, the cells were treated with IL-13 for 24 h.

### 5-ethynyl-2′-deoxyuridine (EdU) assay

Next, we analyzed changes in cell viability in the asthma cell model using the EdU assay. Cell proliferation was measured using the EdU Staining Proliferation Kit (E-CK-A376; Elabscience, China) according to the manufacturer’s instructions. After seeding in 4-well plates, 16HBE cells were incubated with 20-µM EdU for 72 h. The cells were then fixed with 4% paraformaldehyde (P0099; Beyotime, China) following a 2-h incubation. The ratio of EdU-positive cells was used as a quantitative measure of proliferation. Fluorescence intensity in the DAPI and GFP channels was quantified using ImageJ software (CA, US).

### Enzyme-linked immunosorbent assay (ELISA)

ELISA was used to assess changes in inflammation levels in the asthma cell model by measuring the concentrations of human interleukin-18 (IL-18; RX106154H, Ruixin Bio, China), interleukin-1β (IL-1β; RX106152H, Ruixin Bio, China), and interleukin-6 (IL-6; RX106126H, Ruixin Bio, China) in cell culture supernatants. Absorbance at 450 nm was measured using a microplate reader.

### Glutathione (GSH) measurement

A microplate-based reduced GSH assay kit (A006-2-1, Jiancheng Bio, China) was used to measure GSH levels. Reagents and GSH standard samples were prepared according to the manufacturer’s instructions. After centrifuging the cells in a homogenization buffer, the supernatant was mixed with the standard samples and assay reagents. Absorbance was then measured at 405 nm using a microplate reader.

### Superoxide dismutase (SOD) activity assay

SOD levels were measured using a test kit (A001-3, Jiancheng Bio, China) according to the manufacturer’s instructions. Cells were collected via centrifugation and then disrupted by ultrasonication. SOD activity was determined using a microplate reader by measuring absorbance at 450 nm, following the provided guidelines.

### Malondialdehyde (MDA) measurement

The levels of MDA were measured using an MDA detection kit (A003-1, Jiancheng Bio, China). Following the manufacturer’s instructions, a mixed solution was prepared using the kit’s designated chemicals and cell samples. After incubation, the samples were chilled and centrifuged at 3500 rpm. Absorbance was then measured at 532 nm using a microplate reader.

### Ferrous (Fe^2^^+^) and ferric (Fe^3^^+^) ion measurement

We measured Fe^2^^+^ and Fe^3^^+^ levels using a colorimetric iron assay kit (I291, Tongren Chemical, China). After cell lysis on ice, the samples were centrifuged at 15,000 × g. An iron-reducing reagent was added to the supernatant, followed by a 40-min incubation. Optical density at 593 nm was then measured using a microplate reader.

### Reactive oxygen species (ROS) measurement

Using a ROS detection kit (BB-47053, Beibo, China), RO levels were measured. Cells were washed with phosphate-buffered saline (PBS; C0221A, Beyotime, China) after centrifugation and pellet collection. DCFH-DA was diluted (1:1000) in serum-free medium, and the cells were incubated in the dark at 37 ^∘^C for 30 min. After incubation, the cells were rinsed with a serum-free medium. Finally, fluorescence images were captured using an inverted fluorescence microscope.

### Quantitative real-time polymerase chain reaction (qRT-PCR)

Total RNA was extracted using TRIzol reagent (15596018CN, Thermo Fisher Scientific, USA). RNA quality and concentration were assessed with a NanoDrop spectrophotometer (Thermo Fisher Scientific, USA) at 260/280 nm. Reverse transcription was performed using a reverse transcription kit with dsDNase (BL699A, Biosharp, China). Quantitative PCR was conducted on a Bio-Rad fluorescent real-time PCR system (Bio-Rad, USA) using 2× SYBR Green qPCR Master Mix (High ROX) (MPC2208011, Servicebio, China). The relative mRNA levels of target genes were determined using the 2^−ΔΔCt^ method, with β-actin as the internal control. A list of primers is provided in Table S1.

### Western blot

After collecting the cells, they were lysed on ice for 30 min in the lysis buffer (BL504A, Biosharp, China). The lysates were then centrifuged at 12,000 rpm for 15 min, and the resulting supernatant, containing total cell protein, was collected. Proteins were separated by sodium dodecyl sulfate-polyacrylamide gel electrophoresis (SDS-PAGE) (10%, Servicebio, China) and transferred onto polyvinylidene fluoride (PVDF) membranes (IPVH00010, Millipore, Germany). The membranes were incubated overnight at 4 ^∘^C with the appropriate primary antibodies (Table S2). After three washes with TBST, they were incubated for 2 h at room temperature with either goat anti-mouse IgG (H + L) (ZB-2305, Zs-BIO, China) or goat anti-rabbit IgG (H + L) HRP (ZB-2301, Zs-BIO, China). Protein bands were visualized using a chemiluminescent reagent (BL520B, Biosharp, China) and quantified with ImageJ software.

### Transmission electron microscopy (TEM)

TEM was used to examine alterations in mitochondrial function and cell morphology. After trypsinization, the cells were centrifuged and washed with PBS. They were then fixed in 4% paraformaldehyde and dehydrated using a graded ethanol series (64-17-5, Nanjing Reagent, China). The samples were embedded in epoxy resin (45359-1EA-F, Merck, USA) and sectioned to a thickness of 75 nm. The sections were placed on carbon-coated copper grids and stained with uranyl acetate (SPI-02624, HEAD, China) for 10 min, followed by lead citrate (HD17800, HEAD, China) for an additional 10 min. TEM (CM-100, Philips, Netherlands) was used to analyze the samples. Ultrastructural changes in mitochondria observed via TEM serve as a crucial indicator for determining whether ferroptosis (iron-dependent cell death) occurs in cells.

### Statistical analysis

The structural data obtained from the experimental analysis were statistically evaluated for significance and reliability. Pearson correlation was used for correlation analysis. ROC curves were plotted using the survival ROC program. Statistical analysis was performed using GraphPad Prism 8.2.0. Group comparisons were conducted using Student’s *t*-test or one-way analysis of variance (ANOVA) with Tukey’s post hoc test. Results are presented as mean ± standard deviation, with a *P* value of < 0.05 considered statistically significant.

## Results

### IL-13-induced ferroptosis in 16HBE cells

**Figure 1.**
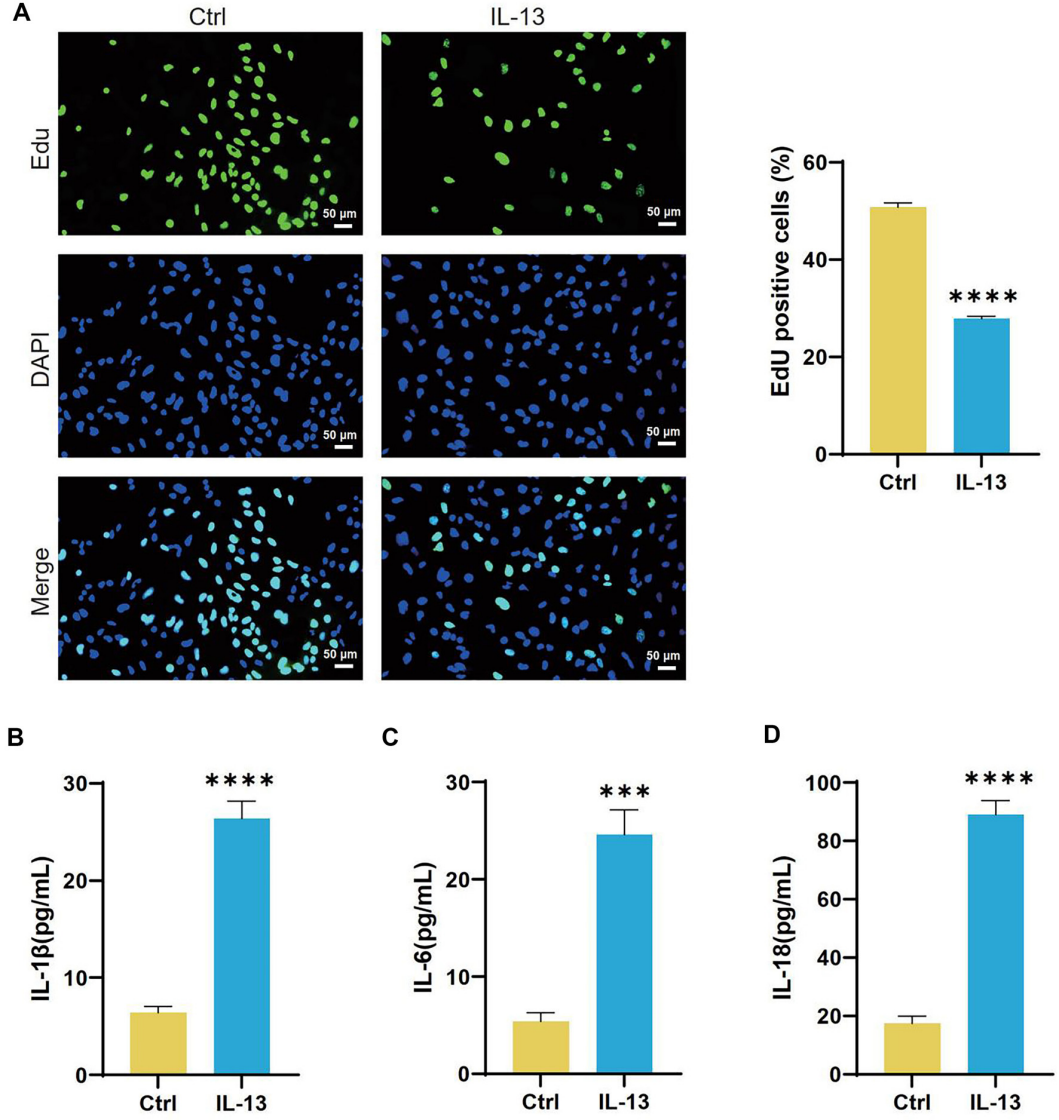
**IL-13 inhibits 16HBE cell proliferation and induces inflammation.** (A) Representative results and quantitative analysis of 5-ethynyl-2’-deoxyuridine (EDU) staining in 16HBE cells from the Control (Ctrl) and IL-13 groups. EDU-positive cells (green) are labeled, and cell nuclei are stained with 4’,6-diamidino-2-phenylindole (DAPI) (blue); (B–D) ELISA measurements of IL-1β (B), IL-6 (C), and IL-18 (D) levels in 16HBE cells from the Ctrl and IL-13 groups. ***P < 0.001, ****P < 0.0001. IL-1β: Interleukin-1β; IL-6: Interleukin-6; IL-18: Interleukin-18; IL-13: Interleukin-13; ELISA: Enzyme-linked immunosorbent assay.

**Figure 2.**
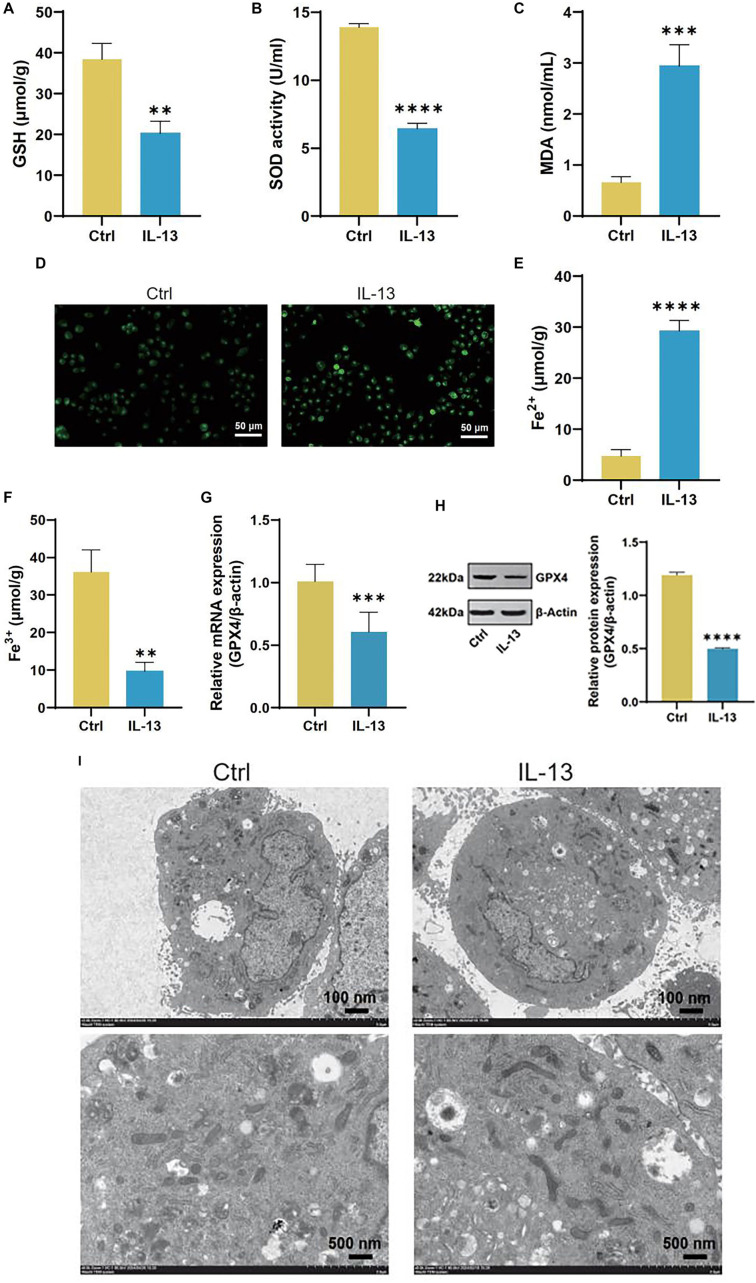
**IL-13 induces ferroptosis in 16HBE cells.** (A–F) Assay kit measurements of glutathione (GSH) (A), SOD (B), MDA (C), ROS (D), Fe^2+^ (E), and Fe^3+^ (F) levels in 16HBE cells from the Ctrl and IL-13 groups; (G) qRT-PCR analysis of glutathione peroxidase 4 (GPX4) mRNA levels in 16HBE cells from the Ctrl and IL-13 groups; (H) Western blotting (WB) analysis of GPX4 protein levels in 16HBE cells from the Ctrl and IL-13 groups; (I) TEM images showing mitochondrial morphology in 16HBE cells from the Ctrl and IL-13 groups. **P < 0.01, ***P < 0.001, ****P < 0.0001. SOD: Superoxide dismutase; MDA: Malondialdehyde; ROS: Reactive oxygen species; qRT-PCR: Quantitative real-time polymerase chain reaction; TEM: Transmission electron microscopy; IL-13: Interleukin-13.

In the 16HBE cell line, IL-13 treatment significantly inhibited cell proliferation compared to the control group (Figure 1A). Following IL-13 exposure, levels of IL-1β, IL-6, and IL-18 increased substantially (Figure 1B–1D). Additionally, IL-13 treatment resulted in elevated MDA and ROS levels, along with reduced SOD and GSH levels (Figure 2A–2D). Analysis of iron content in 16HBE cells post-IL-13 treatment revealed an increase in Fe^2^^+^ levels and a decrease in Fe^3^^+^ levels compared to the control group (Figure 2E and 2F). Furthermore, IL-13 downregulated the ferroptosis marker GPX4 (Figure 2G and 2H). Electron microscopy showed ferroptosis-related mitochondrial abnormalities, including mitochondrial shrinkage, reduced cristae, and outer membrane rupture (Figure 2D).

### Functional enrichment analysis of DEGs

**Figure 3.**
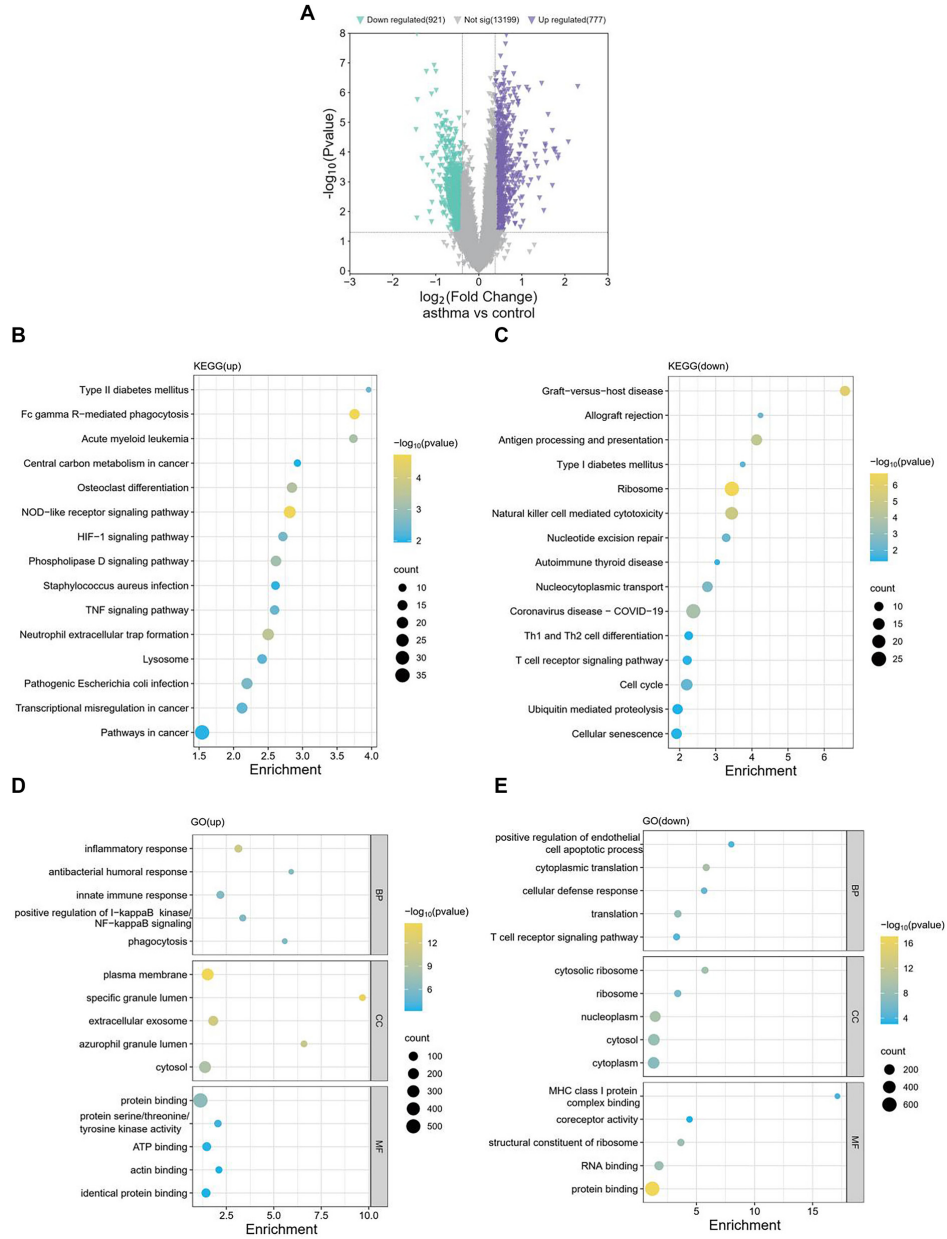
**Differential expression analysis and functional enrichment analysis.** (A) Volcano plot of DEGs in asthma from the GSE134544 dataset. Purple represents upregulated genes, and green represents downregulated genes; (B) Kyoto Encyclopedia of Genes and Genomes (KEGG) enrichment analysis of upregulated DEGs; (C) KEGG enrichment analysis of downregulated DEGs; (D) GO enrichment analysis of upregulated DEGs; (E) GO enrichment analysis of downregulated DEGs. DEGs: Differentially expressed genes; GO: Gene Ontology.

To explore the molecular mechanisms of asthma, 1698 DEGs were identified from the GSE134544 dataset, including 777 upregulated and 921 downregulated genes (Figure 3A). KEGG pathway and GO enrichment analyses were conducted to uncover the potential biological functions of these DEGs. Upregulated DEGs were enriched in pathways, such as the TNF signaling pathway, HIF-1 signaling pathway, and NOD-like receptor signaling pathway (Figure 3B). KEGG enrichment analysis also revealed that downregulated DEGs were significantly associated with pathways including cellular senescence, the T cell receptor signaling pathway, and Th1/Th2 cell differentiation (Figure 3C). For BP, upregulated DEGs were primarily involved in IκB kinase/NF-κB signaling, immune response, and inflammation (Figure 3D). In terms of CC, they were linked to the cytosol, exosomes, and plasma membrane. Regarding MF, they were associated with protein binding, actin binding, ATP binding, and kinase activity (Figure 3D). Downregulated DEGs in BP were related to T cell receptor signaling, translation, and cellular defense (Figure 3E). In CC, they were predominantly found in the cytoplasm, nucleoplasm, and ribosomes. For MF, they were enriched in protein and RNA binding, ribosomal structure, and MHC class I binding (Figure 3E). These findings suggest that DEGs may contribute to asthma development through these gene-regulatory pathways.

### WGCNA analysis and key module identification

**Figure 4.**
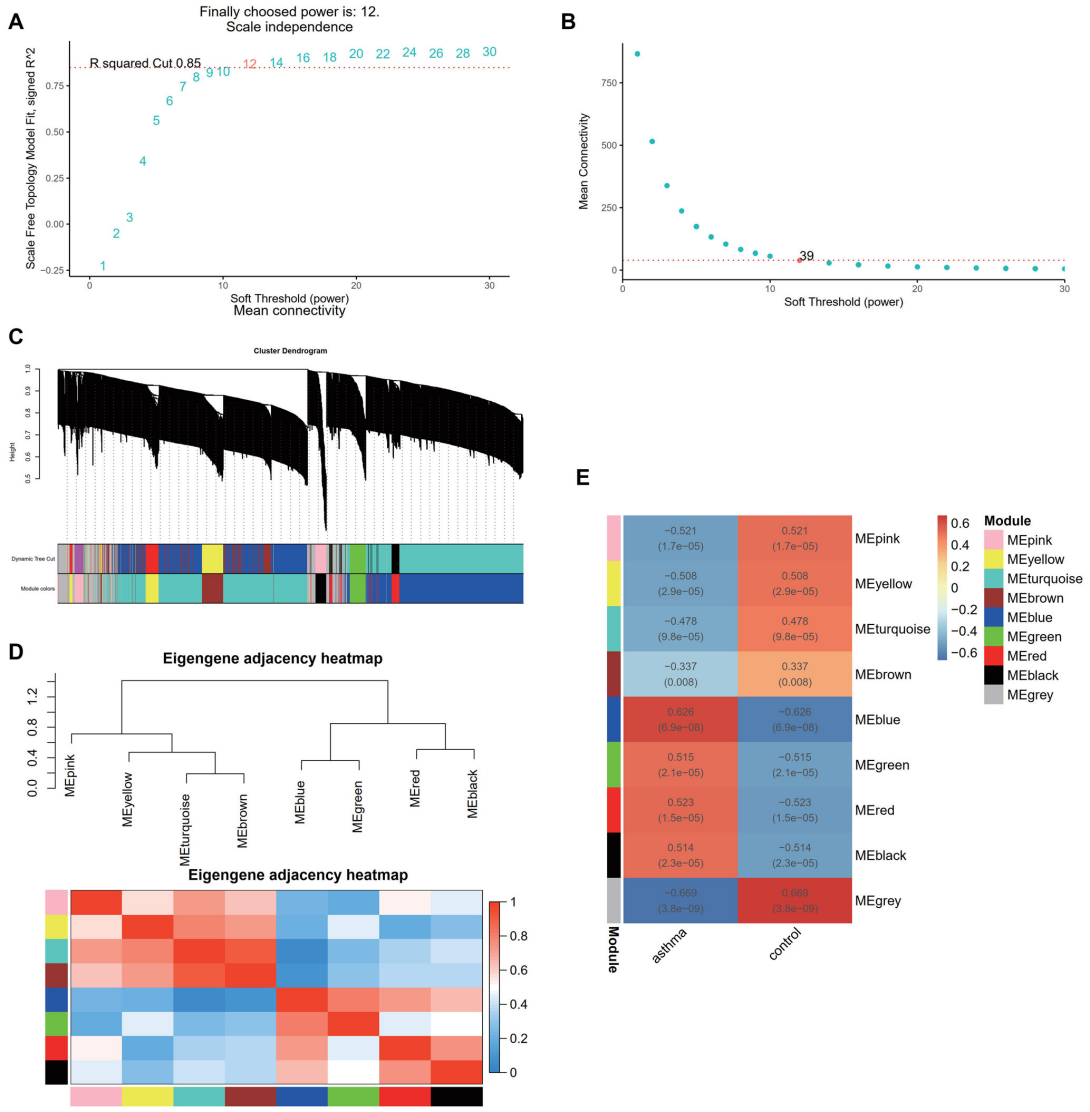
**WGCNA and core module identification.** (A) Analysis of the scale-free index (β) for various soft-threshold powers; (B) Analysis of the mean connectivity for various soft-threshold powers; (C) Gene clustering dendrogram based on topological overlap and assigned module colors; (D) Heatmap of feature gene adjacency for the eight modules; (E) Heatmap showing the correlation between each module’s feature genes and phenotypes. WGCNA: Weighted gene co-expression network analysis.

WGCNA was used to identify key modules associated with asthma. Using a soft threshold of 12 (R^2^ ═ 0.85), we constructed a scale-free network (Figure 4A and 4B). The adjacency matrix was generated, and the TOM was built (Figure 4C). Module reliability was confirmed through correlation analysis (Figure 4D). Further correlation analysis between modules and phenotype data identified the blue module as strongly associated with asthma (Figure 4E). Consequently, we selected the blue module for further analysis.

### Hub gene identification and expression analysis

**Figure 5.**
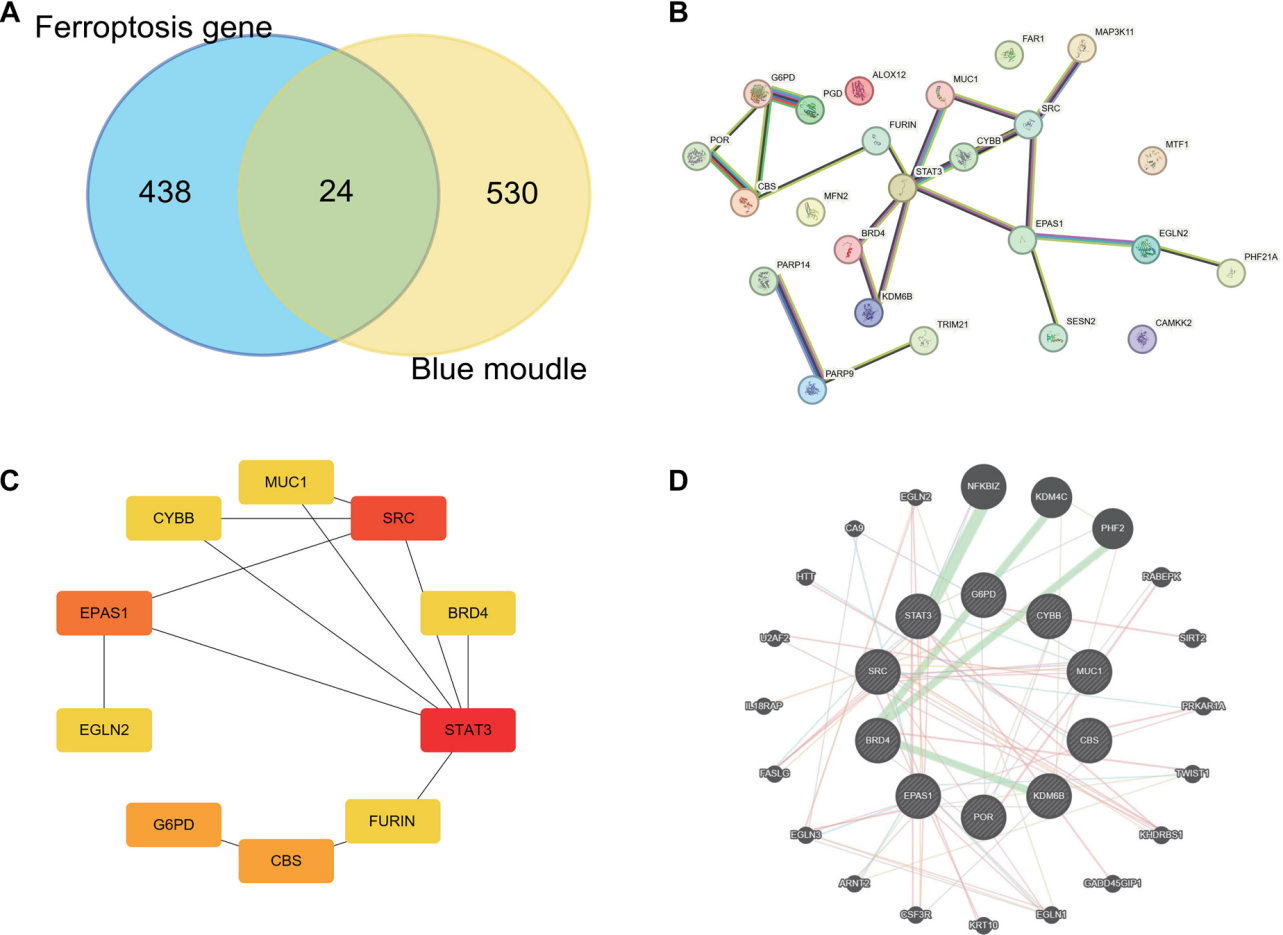
**Interaction network construction and hub gene identification.** (A) Venn diagram showing overlapping genes between asthma-related module genes and ferroptosis-related genes; (B) PPI network of overlapping genes. Edges between nodes represent gene-gene interactions; (C) PPI network of the top 10 hub genes based on degree; (D) Gene–gene interaction network of the top 10 hub genes. PPI: Protein–protein interaction.

**Figure 6.**
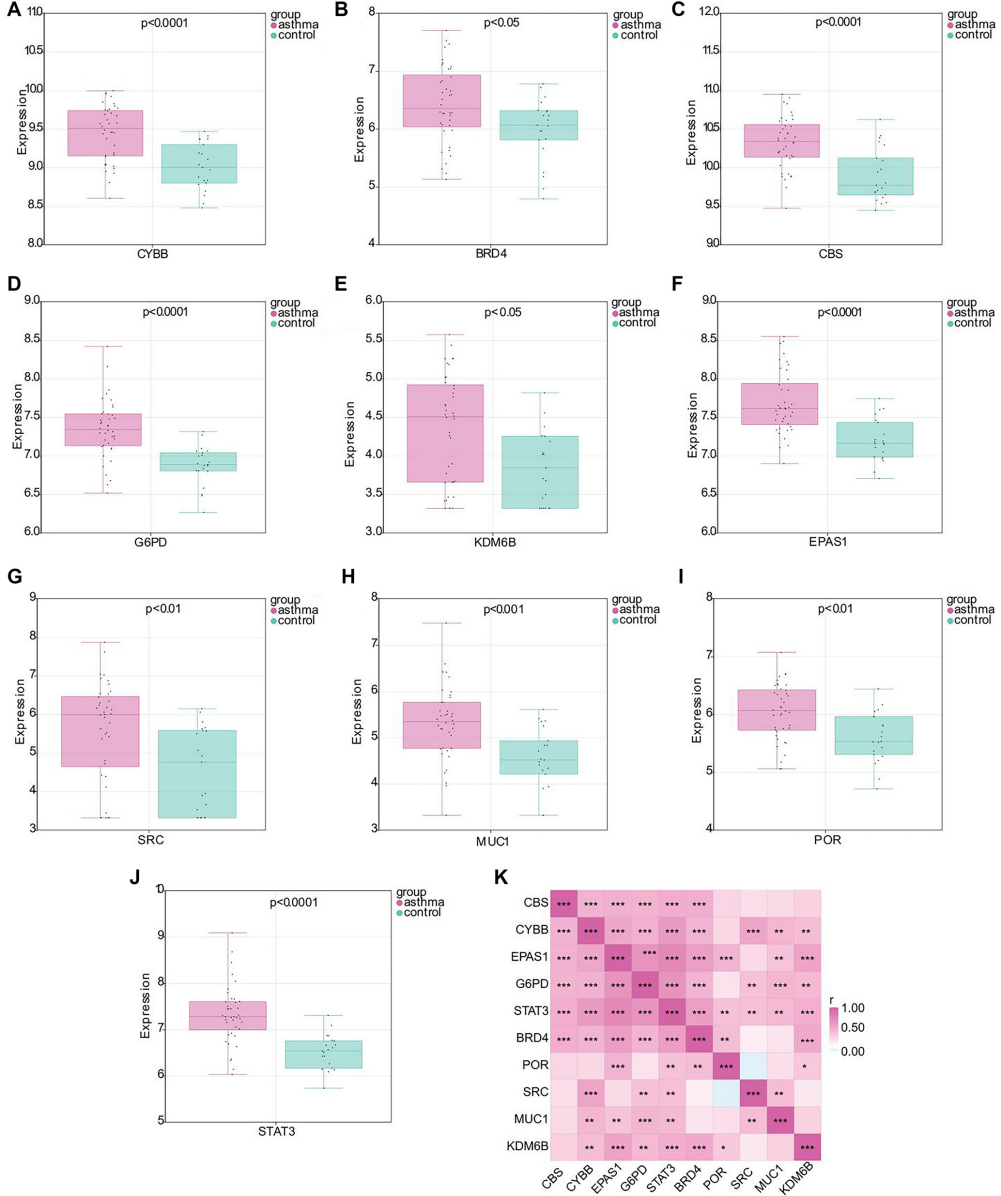
**Expression levels and correlation analysis of hub genes.** Expression levels of CYBB (A), BRD4 (B), CBS (C), G6PD (D), KDM6B (E), EPAS1 (F), SRC (G), MUC1 (H), POR (I), and STAT3 (J) in asthma and control samples based on the GSE134544 database; (K) Pearson correlation analysis of the relationships between hub genes. ^*^P < 0.05, **P < 0.01, ***P < 0.001. EPAS1: Endothelial PAS Domain Protein 1; STAT3: Signal transducer and activator of transcription 3.

To investigate the involvement of ferroptosis in asthma, we identified 472 ferroptosis-related genes from the FerrDb V2 database. Through WGCNA analysis, we extracted 24 ferroptosis-related genes from the blue module (Figure 5A). A PPI network was constructed to explore interactions among these 24 genes, consisting of 24 nodes and 22 edges (Figure 5B). Figure 5C visually represents the network relationships and degree values of the top 10 key genes, with darker colors indicating higher values. These key genes include CYBB, EPAS1, CBS, G6PD, MUC1, BRD4, KDM6B, POR, SRC, and STAT3. Additionally, the gene–gene interaction network highlights the top 20 most frequently altered genes associated with the hub genes (Figure 5D). To assess the expression levels of these key genes, we utilized the GSE134544 database, which revealed significant upregulation of CYBB, EPAS1, CBS, G6PD, MUC1, BRD4, KDM6B, POR, SRC, and STAT3 in asthma patients compared to the control group (Figure 6A–6J). Furthermore, we analyzed the expression correlations among these key genes (Figure 6K).

### ROC and GSEA analysis of hub genes

**Figure 7.**
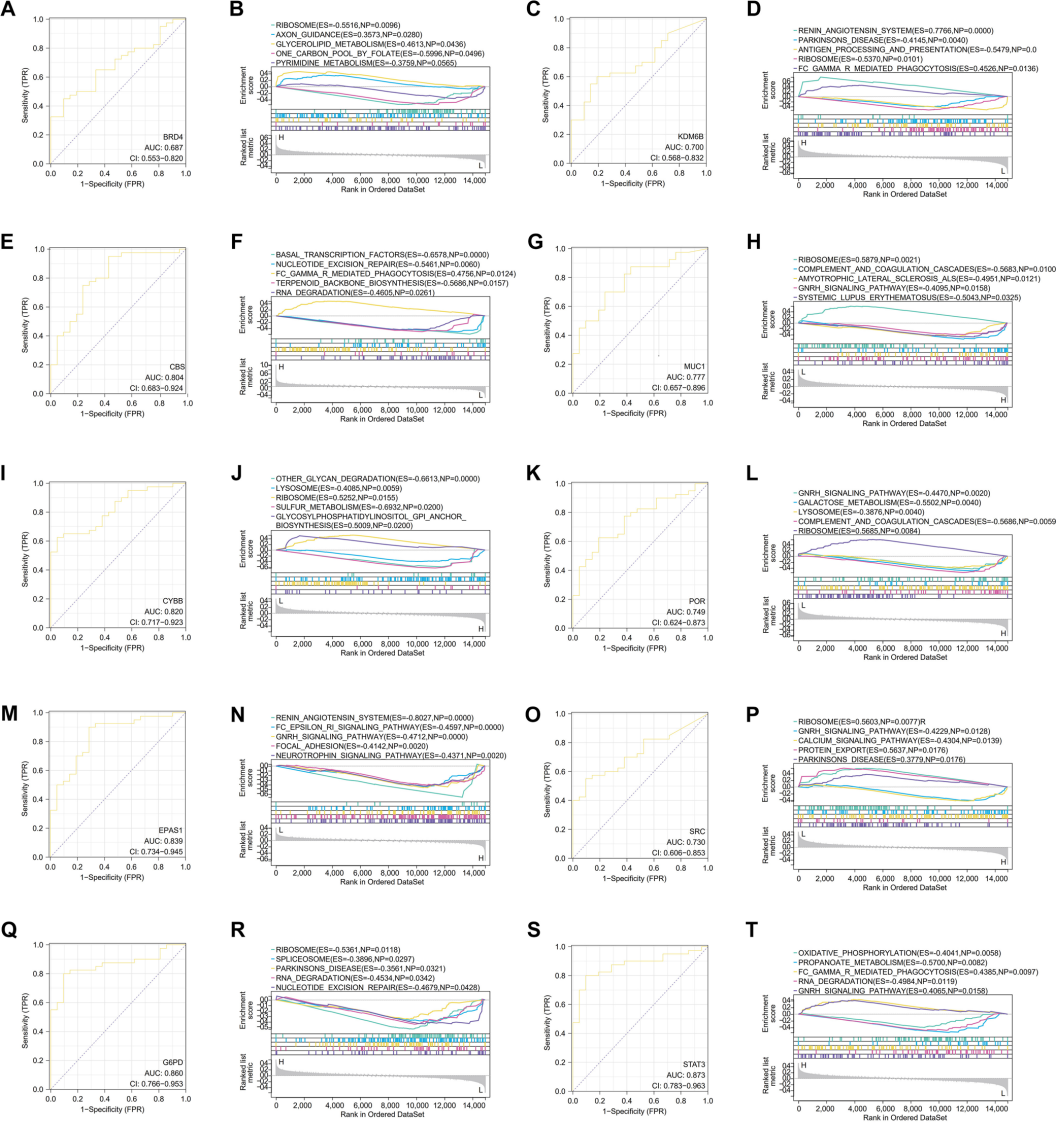
**ROC and GSEA analysis of hub genes.** (A and B) ROC and GSEA of BRD4; (C and D) ROC and GSEA of KDM6B; (E and F) ROC and GSEA of CBS; (G and H) ROC and GSEA of MUC1; (I and J) ROC and GSEA of CYBB; (K and L) ROC and GSEA of POR; (M and N) ROC and GSEA of EPAS1; (O and P) ROC and GSEA of SRC; (Q and R) ROC and GSEA of G6PD; (S and T) ROC and GSEA of STAT3. ROC: Receiver operating characteristic; EPAS1: Endothelial PAS domain protein 1; STAT3: Signal transducer and activator of transcription 3; GSEA: Gene set enrichment analysis.

The area under the ROC curve (AUC) is used to assess the clinical diagnostic value of genes. As shown in Figure 7, CYBB (AUC ═ 0.82, Figure 7I), EPAS1 (AUC ═ 0.839, Figure 7M), CBS (AUC ═ 0.804, Figure 7E), G6PD (AUC ═ 0.86, Figure 7Q), and STAT3 (AUC ═ 0.873, Figure 7S) demonstrate high diagnostic accuracy for asthma (AUC > 0.8). In contrast, MUC1 (AUC ═ 0.777, Figure 7G), BRD4 (AUC ═ 0.687, Figure 7A), KDM6B (AUC ═ 0.7, Figure 7C), POR (AUC ═ 0.749, Figure 7K), and SRC (AUC ═ 0.73, Figure 7O) exhibit lower predictive value. GSEA revealed significant enrichment of BRD4 in ribosome, axon guidance, and glycerolipid metabolism (Figure 7B). KDM6B was enriched in antigen processing, presentation, and FC gamma receptor-mediated phagocytosis (Figure 7D), while CBS showed enrichment in FC gamma receptor-mediated phagocytosis and RNA degradation (Figure 7F). MUC1 was associated with ribosome and the GnRH signaling pathway (Figure 7H), CYBB with ribosome and sulfur metabolism (Figure 7J), and POR with the GnRH signaling pathway and ribosome (Figure 7L). EPAS1 was enriched in the Fc epsilon RI and GnRH signaling pathways (Figure 7N), SRC in the GnRH and calcium signaling pathways (Figure 7P), G6PD in ribosome and nucleotide excision repair (Figure 7R), and STAT3 in oxidative phosphorylation and FC gamma receptor-mediated phagocytosis (Figure 7T). In conclusion, CYBB, EPAS1, CBS, G6PD, and STAT3 demonstrate strong diagnostic potential for asthma.

### Correlation analysis of immune infiltration in asthma

**Figure 8.**
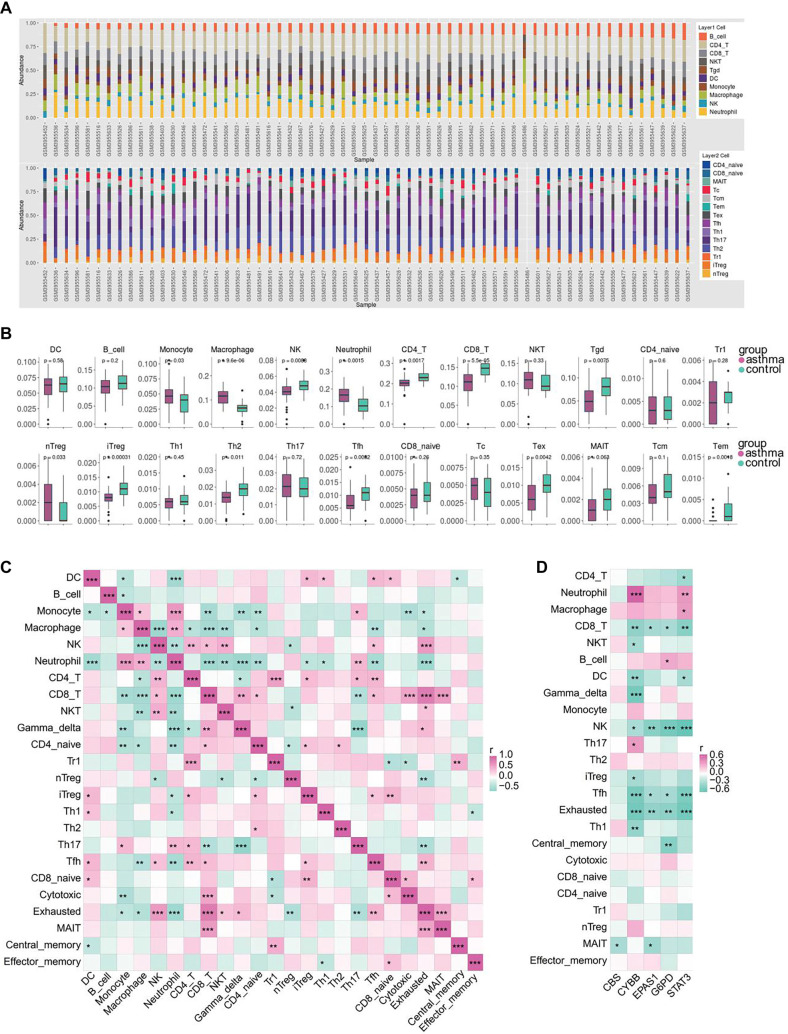
**Immune cell composition landscape in asthma.** (A) Stacked bar chart showing the proportions of various immune cell types in asthma based on ImmuCellAI analysis; (B) Analysis of immune cell levels between asthma (red) and control (green) samples; (C) Heatmap showing correlations between immune cell levels in asthma samples; (D) Heatmap showing correlations between immune cell levels and hub genes in asthma samples.

To evaluate changes in the immune microenvironment in asthma, we conducted an immune analysis using the GSE134544 dataset. We examined the infiltration of 24 immune cell types (Figure 8A), identifying significant differences in 13 of them between asthma and control groups. Asthma samples showed elevated levels of monocytes, macrophages, neutrophils, and nTreg cells, while the control group had higher levels of NK cells, CD4+ T cells, CD8+ T cells, gamma-delta T cells, iTreg cells, Th2 cells, Tfh cells, exhausted T cells, and effector memory T cells (Figure 8B). Further analysis of immune cell infiltration revealed various correlations (Figure 8C). The strongest positive associations were observed between CD8+ T cells and exhausted T (Tex) cells (0.82), neutrophils and monocytes (0.61), and Tex cells and NK cells (0.60). In contrast, the most pronounced negative correlations were found between gamma–delta T (Tgd) cells and Th17 cells (−0.63), Tgd cells and neutrophils (−0.57), and neutrophils and CD8+ T cells (−0.55). Figure 8D illustrates the relationships between immune cells and key genes. CBS was negatively associated with MAIT cells. CYBB showed a positive correlation with Th17 cells and neutrophils but a negative correlation with Th1 cells, exhausted T cells, Tfh cells, iTreg cells, NK cells, Tgd cells, DC cells, NKT cells, and CD8+ T cells. EPAS1 was negatively associated with MAIT cells, Tfh cells, Tex cells, CD8+ T cells, and NK cells. G6PD was negatively correlated with Tcm cells, Tex cells, Tfh cells, NK cells, and CD8+ T cells but positively linked to B cells. STAT3 was negatively associated with Tex cells, Tfh cells, DC cells, NK cells, CD4+ T cells, and CD8+ T cells, while positively correlated with macrophages and neutrophils. These findings suggest that key ferroptosis-related genes may regulate asthma by modulating immune cell infiltration.

### JAK2/STAT3 regulates ferroptosis and inflammatory reaction induced by IL-13 in 16HBE cells

**Figure 9.**
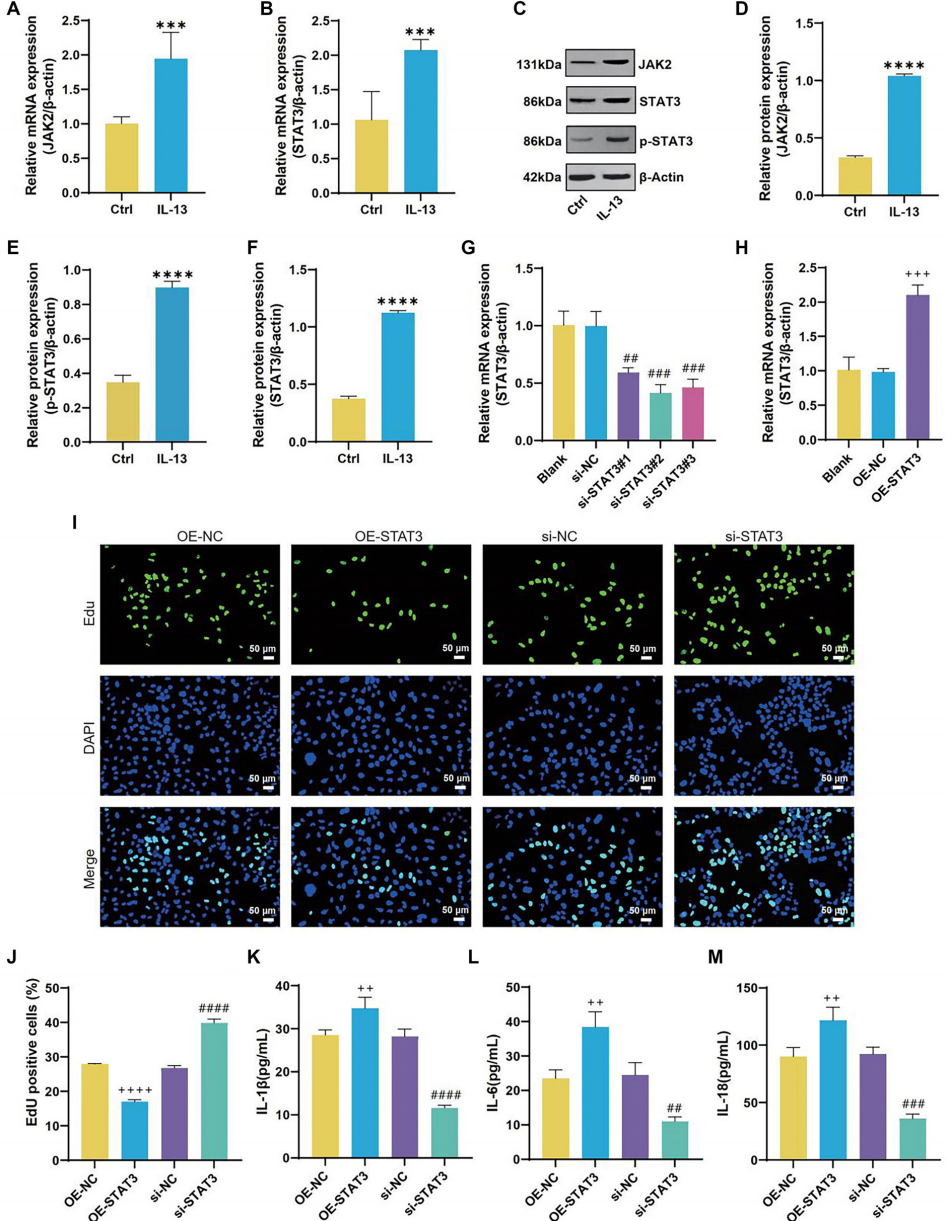
**JAK2/STAT3 involvement in IL-13 regulation of 16HBE cell proliferation and inflammation.** (A and B) QRT-PCR analysis of JAK2 and STAT3 mRNA levels in 16HBE cells from the Control (Ctrl) and IL-13 groups; (C–F) Western blot (WB) analysis of JAK2, phosphorylated STAT3 (p-STAT3), and STAT3 protein levels in 16HBE cells from the Ctrl and IL-13 groups; (G and H) qRT-PCR analysis of transfection efficiency for siRNA targeting STAT3 and overexpression plasmids; (I and J) Representative results and quantitative analysis of EDU staining in 16HBE cells from OE-NC, OE-STAT3, si-NC, and si-STAT3 groups. EDU-positive cells (green) are labeled, and cell nuclei are stained with DAPI (blue); (K–M) ELISA measurements of IL-1β (K), IL-6 (L), and IL-18 (M) in 16HBE cells from OE-NC, OE-STAT3, si-NC, and si-STAT3 groups. ***P < 0.001, ****P < 0.0001 compared to Ctrl group; ^##^*P* < 0.01, ^###^*P* < 0.001, ^####^*P* < 0.0001 compared to si-NC group; ^++^*P* < 0.01, ^+++^*P* < 0.001, ^++++^*P* < 0.0001 compared to OE-NC group. IL-13: Interleukin-13; IL-1β: Interleukin-1β; IL-6: Interleukin-6; IL-18: Interleukin-18; JAK2: Janus kinase 2; STAT3: Signal transducer and activator of transcription 3; ELISA: Enzyme-linked immunosorbent assay; qRT-PCR: Quantitative real-time polymerase chain reaction.

**Figure 10.**
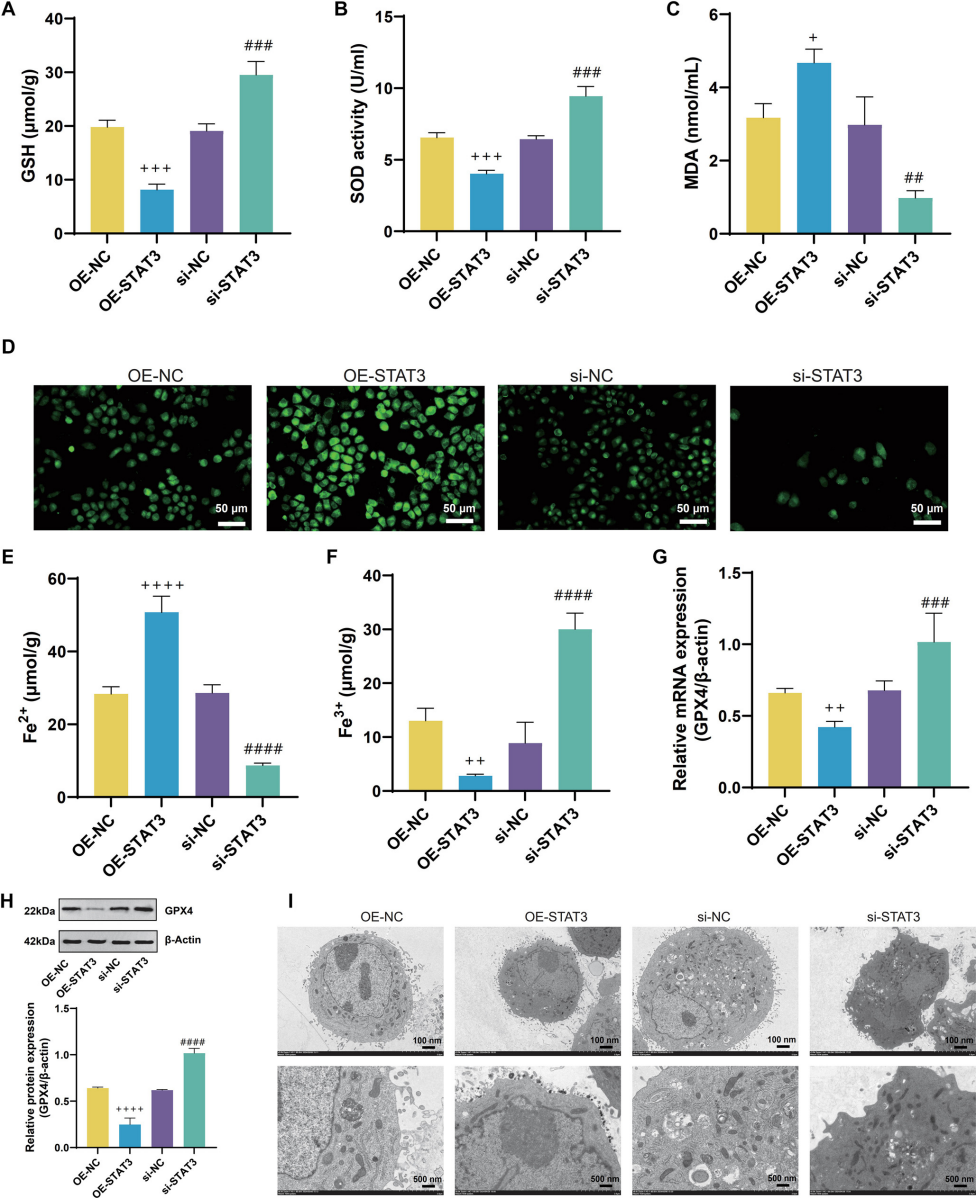
**IL-13 induces ferroptosis in 16HBE cells via JAK2/STAT3.** (A–F) Measurement of GSH (A), SOD (B), MDA (C), ROS (D), Fe^2^^+^ (E), and Fe^3^^+^ (F) levels in 16HBE cells from OE-NC, OE-STAT3, si-NC, and si-STAT3 groups using assay kits; (G) QRT-PCR analysis of GPX4 mRNA levels in 16HBE cells from OE-NC, OE-STAT3, si-NC, and si-STAT3 groups; (H) WB analysis of GPX4 protein levels in 16HBE cells from OE-NC, OE-STAT3, si-NC, and si-STAT3 groups; (I) TEM analysis of mitochondrial morphology in 16HBE cells from OE-NC, OE-STAT3, si-NC, and si-STAT3 groups.^##^*P* < 0.01, ^###^*P* < 0.001, ^####^*P* < 0.0001 compared to si-NC group. ^+^*P* < 0.05, ^++^*P* < 0.01, ^+++^*P* < 0.001, ^++++^*P* < 0.0001 compared to OE-NC group. IL-13: Interleukin-13; JAK2: Janus kinase 2; STAT3: Signal transducer and activator of transcription 3; TEM: Transmission electron microscopy; ROS: Reactive oxygen species; MDA: Malondialdehyde; SOD: Superoxide dismutase.

Bioinformatics analyses identified STAT3 as a key ferroptosis-related gene implicated in asthma. Prior research has shown that JAK2/STAT3 signaling profoundly influences inflammatory and immune responses in various disease contexts. Here, qRT-PCR confirmed that IL-13 stimulation upregulated JAK2 and STAT3 mRNA levels in 16HBE cells (Figure 9A and 9B). Consistent with these findings, Western blot analyses revealed elevated JAK2, total STAT3, and p-STAT3 upon IL-13 treatment (Figure 9C–9F). To probe STAT3 function, 16HBE cells were transfected with either siRNA targeting STAT3 or an overexpression plasmid (Figure 9G and 9H). Among the siRNAs tested, si-STAT3#2 achieved the highest knockdown efficiency and was selected for subsequent experiments. EdU assays showed that STAT3 overexpression significantly reduced cell viability, whereas STAT3 knockdown markedly enhanced it (Figure 9I and 9J). Furthermore, ELISA revealed that STAT3 promoted the production of proinflammatory cytokines—IL-6, IL-18, and IL-1β—in IL-13-treated 16HBE cells (Figure 9K–9M). Additional assays demonstrated that STAT3 inhibition reduced GSH, SOD, Fe^3^^+^, and GPX4, while increasing MDA, ROS, and Fe^2^^+^ in IL-13-induced 16HBE cells (Figure 10A–10H). Electron microscopy (Figure 10I) further corroborated these effects: the OE-STAT3 group displayed profound mitochondrial alterations—outer membrane rupture, fewer cristae, and mitochondrial shrinkage—whereas STAT3 knockdown mitigated these morphological changes. Collectively, these results underscore the pivotal role of STAT3 in driving ferroptosis and inflammatory responses in IL-13-treated 16HBE cells, offering new insights into the pathogenesis of asthma.

### IL-13 upregulates EPAS1 expression via JAK2/STAT3 to regulate ferroptosis and inflammatory reaction in 16HBE cells

**Figure 11.**
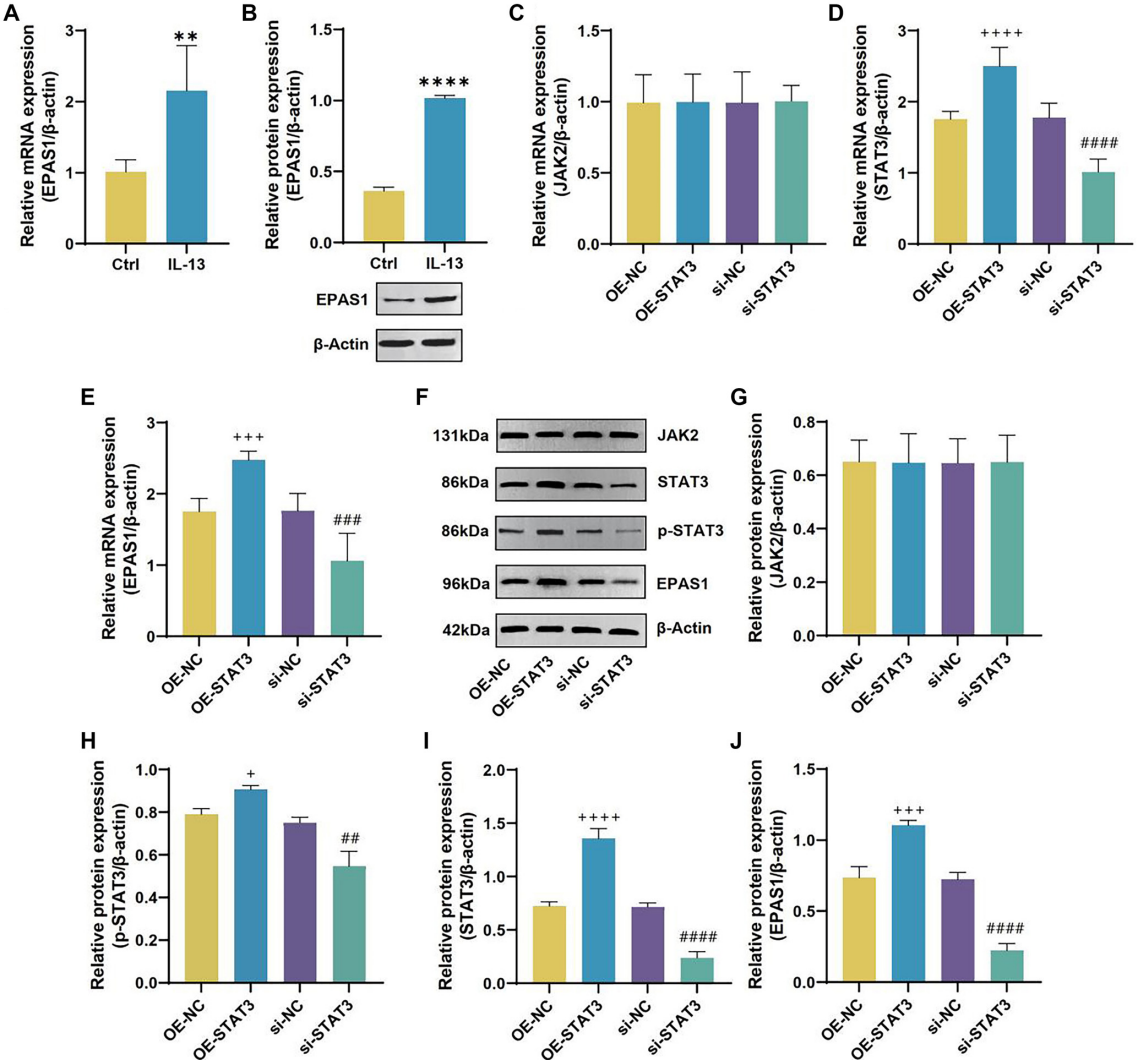
**IL-13 upregulates EPAS1 via JAK2/STAT3.** (A) QRT-PCR analysis of EPAS1 mRNA levels in 16HBE cells from Ctrl and IL-13 groups; (C–E) QRT-PCR analysis of JAK2 (C), STAT3 (D), and EPAS1 (E) mRNA levels in 16HBE cells from OE-NC, OE-STAT3, si-NC, and si-STAT3 groups; (F and G) WB analysis of JAK2 (G), p-STAT3 (H), STAT3 (I), and EPAS1 (J) protein levels in 16HBE cells from OE-NC, OE-STAT3, si-NC, and si-STAT3 groups.**P < 0.01, ****P < 0.0001 compared to Ctrl group. ^##^*P* < 0.01, ^###^*P* < 0.001, ^####^*P* < 0.0001 compared to si-NC group. ^+^*P* < 0.05, ^+++^*P* < 0.001, ^++++^*P* < 0.0001 compared to OE-NC group. EPAS1: Endothelial PAS Domain Protein 1; IL-13: Interleukin-13; JAK2: Janus kinase 2; STAT3: Signal transducer and activator of transcription 3.

**Figure 12.**
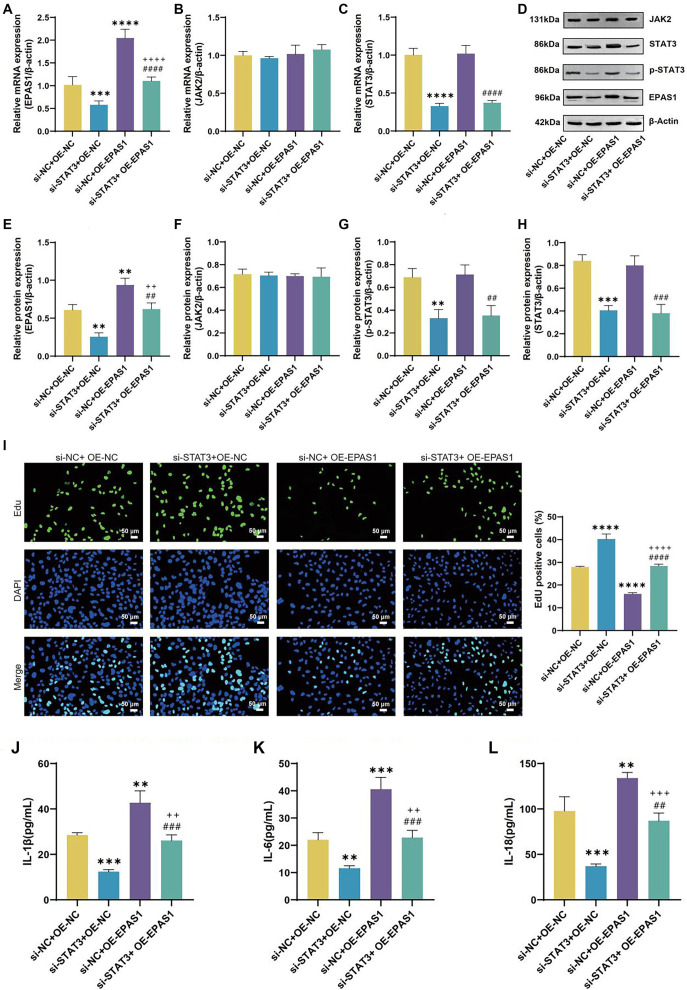
**IL-13 regulates 16HBE cell proliferation and inflammation via JAK2/STAT3-mediated upregulation of EPAS1.** (A–C) qRT-PCR analysis of EPAS1 (A), JAK2 (B), and STAT3 (C) mRNA levels in 16HBE cells from si-NC+OE-NC, si-STAT3+OE-NC, si-NC+OE-EPAS1, and si-STAT3+OE-EPAS1 groups; (D–H) WB analysis of EPAS1 (E), JAK2 (F), p-STAT3 (G), and STAT3 (H) protein levels in 16HBE cells from OE-NC, OE-STAT3, si-NC, and si-STAT3 groups; (I) Representative results and quantitative analysis of EDU staining in 16HBE cells from si-NC+OE-NC, si-STAT3+OE-NC, si-NC+OE-EPAS1, and si-STAT3+OE-EPAS1 groups. EDU-positive cells (green) are labeled, and cell nuclei are stained with DAPI (blue); (J–L) ELISA measurements of IL-1β (J), IL-6 (K), and IL-18 (L) in 16HBE cells from si-NC+OE-NC, si-STAT3+OE-NC, si-NC+OE-EPAS1, and si-STAT3+OE-EPAS1 groups.**P < 0.01, ***P < 0.001, ****P < 0.0001 compared to si-NC+OE-NC group. ^+++^*P* < 0.001, ^++++^*P* < 0.0001 compared to si-STAT3+OE-NC group. ^##^*P* < 0.01, ^###^*P* < 0.001, ^####^*P* < 0.0001 compared to si-NC+OE-EPAS1 group. EPAS1: Endothelial PAS Domain Protein 1; IL-13: Interleukin-13; JAK2: Janus kinase 2; STAT3: Signal transducer and activator of transcription 3; ELISA: Enzyme-linked immunosorbent assay; qRT-PCR: Quantitative real-time polymerase chain reaction.

**Figure 13.**
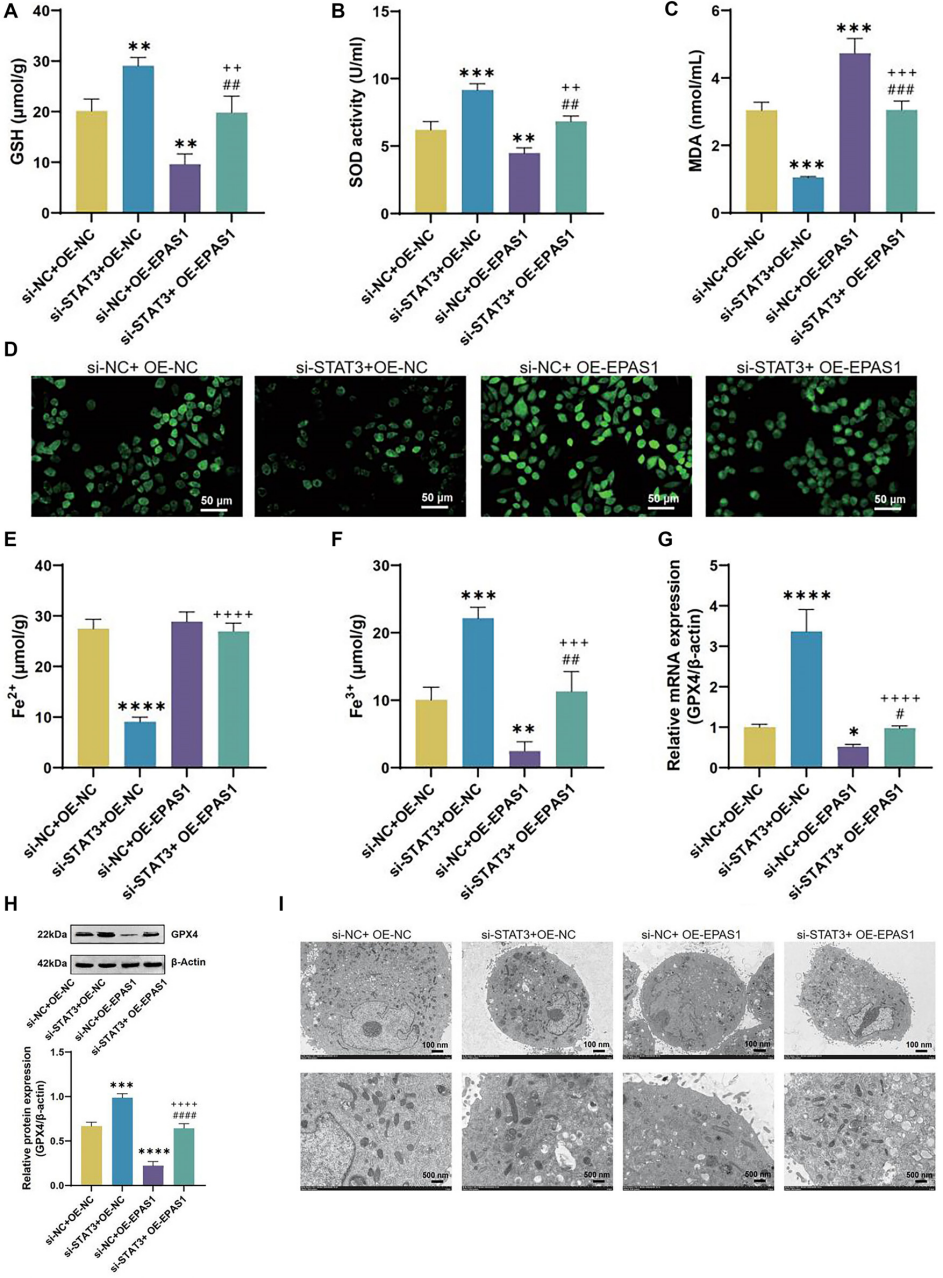
**IL-13-induced ferroptosis in 16HBE cells regulated by EPAS1 upregulation via JAK2/STAT3**. (A–C) Measurement of GSH (A), SOD (B), MDA (C), ROS (D), Fe^2^^+^ (E), and Fe^3^^+^ (F) levels in 16HBE cells from si-NC+OE-NC, si-STAT3+OE-NC, si-NC+OE-EPAS1, and si-STAT3+OE-EPAS1 groups using assay kits; (G) qRT-PCR analysis of GPX4 mRNA levels in 16HBE cells from si-NC+OE-NC, si-STAT3+OE-NC, si-NC+OE-EPAS1, and si-STAT3+OE-EPAS1 groups; (H) WB analysis of GPX4 protein levels in 16HBE cells from si-NC+OE-NC, si-STAT3+OE-NC, si-NC+OE-EPAS1, and si-STAT3+OE-EPAS1 groups; (I) TEM analysis of mitochondrial morphology in 16HBE cells from si-NC+OE-NC, si-STAT3+OE-NC, si-NC+OE-EPAS1, and si-STAT3+OE-EPAS1 groups.^*^P < 0.05, **P < 0.01, ***P < 0.001, ****P < 0.0001 compared to si-NC+OE-NC group. ^++^*P* < 0.01, ^+++^*P* < 0.001, ^++++^*P* < 0.0001 compared to si-STAT3+OE-NC group. ^#^*P* < 0.05, ^##^*P* < 0.01, ^###^*P* < 0.001, ^####^*P* < 0.0001 compared to si-NC+OE-EPAS1 group. EPAS1: Endothelial PAS Domain Protein 1; IL-13: Interleukin-13; JAK2: Janus kinase 2; STAT3: Signal transducer and activator of transcription 3; TEM: Transmission electron microscopy; qRT-PCR: Quantitative real-time polymerase chain reaction; ROS: Reactive oxygen species; MDA: Malondialdehyde; SOD: Superoxide dismutase.

A number of malignancies are linked to dysregulation of EPAS1 expression, which also controls ferroptosis in clear cell and cervical cancers [20]. IL-13 treatment increased EPAS1 levels (Figure 11A and 11B). Notably, previous PPI network analysis indicated an interaction between STAT3 and EPAS1. We further explored the correlation between STAT3 and EPAS1 expression and found that STAT3 positively regulates EPAS1 expression without affecting JAK2 levels (Figure 11C–11J). We constructed an OE-EPAS1 plasmid for overexpressing EPAS1 in 16HBE cells. Results showed that OE-EPAS1 stopped the decline in EPAS1 levels caused by STAT3 knockdown (Figure 12A, 12D, and 12E). Knockdown of STAT3 or overexpression of EPAS1 did not significantly alter JAK2 expression (Figure 12B, 12D, and 12F). Overexpression of EPAS1 did not significantly affect STAT3 or p-STAT3 levels (Figure 12C, 12D, 12G, and 12H). When 16HBE cells in the si-STAT3+OE-NC group were juxtaposed to the si-NC+OE-NC group, they had higher cell viability and lower levels of IL-1β, IL-6, and IL-18, while the si-NC+OE-EPAS1 group showed the opposite results (Figure 12I–12L). Cell viability in the si-STAT3+OE-EPAS1 group was higher than in the si-NC+OE-EPAS1 group, but lower than in the si-STAT3+OE-NC group (Figure 12I). The si-STAT3+OE-EPAS1 group had higher doses of IL-1β, IL-6, and IL-18 than the si-STAT3+OE-NC group, but not as high as the si-NC+OE-EPAS1 group (Figure 12J–12L). As opposed to si-NC+OE-NC group, the si-STAT3+OE-NC group showed increased levels of GSH, SOD, Fe^3+^, and GPX4, and decreased levels of MDA, ROS, and Fe^2+^, while the si-NC+OE-EPAS1 group exhibited the opposite results (Figure 13A–13H). Lower than in the si-STAT3+OE-NC group, but greater than in the si-NC+OE-EPAS1 group, were the levels of GSH, SOD, Fe3^+^, and GPX4 (Figure 13A–13H). The TME results showed that whereas mitochondrial damage was worsened in the si-NC+OE-EPAS1 group, it was lessened in the si-STAT3+OE-NC group in regard to the si-NC+OE-NC group (Figure 13I). More mitochondrial damage than in the si-STAT3+OE-NC group, but less than in the si-NC+OE-EPAS1 group, was seen in the si-STAT3+OE-EPAS1 group (Figure 13A–13H).

## Discussion

Bioinformatics is a discipline that applies computational techniques and statistical methods to analyze, process, and interpret biological data, aiming to uncover the underlying principles of BPs. In recent years, bioinformatics has played a pivotal role in elucidating molecular disease mechanisms and identifying potential biomarkers through big data analysis. Liao et al. [24] integrated machine learning and bioinformatics to identify ABHD5 as a lipid-related biomarker in idiopathic pulmonary fibrosis. Liu et al. [25] applied bioinformatics and machine learning to identify six immune-related hub genes in atherosclerosis with rheumatoid arthritis. Additionally, Liao et al. [26] combined bioinformatics with experimental validation to demonstrate that SPP1 is a key regulator of the PI3K/AKT signaling pathway in pulmonary fibrosis. Through bioinformatics analysis, we identified 1698 DEGs associated with asthma. These DEGs were highly enriched in Th1 and Th2 cell differentiation, the TNF signaling pathway, and the HIF-1 signaling pathway. Previous studies have shown that vitamin D improves asthma-induced lung damage by modulating the HIF-1α signaling pathway [27]. TNF-α, a key component of the TNF signaling system, plays a crucial role in asthma-related inflammation [28]. Anti-TNF-α treatments have been shown to improve lung function, reduce airway hyperreactivity, and enhance the quality of life in asthma patients, while also decreasing the frequency of acute exacerbations [29]. An imbalance between Th1 and Th2 cells is a major immune abnormality in asthma, with cytokine levels in the serum regulated by Th1/Th2 differentiation in the lungs of asthma patients [30, 31]. These findings underscore the critical roles of Th1/Th2 immune responses, the TNF signaling pathway, and the HIF-1 signaling pathway in asthma pathogenesis, suggesting potential new biomarkers and therapeutic targets for treatment. Our investigation also identified important genes involved in ferroptosis and asthma, including EPAS1, STAT3, G6PD, CYBB, and CBS. CYBB, a subunit of NADPH oxidase, contributes to endogenous oxidative stress [32]. Mycobacterium leprae promotes macrophage ferroptosis through CYBB, aiding its survival [33]. In mesenchymal glioblastoma, CYBB regulates temozolomide (TMZ) resistance via the ferroptosis-regulating Nrf2/SOD2 axis [34]. G6PD, a key enzyme in glycolysis, is essential for generating reduced glutathione and NADPH, both crucial for combating oxidative stress [33]. In hepatocellular carcinoma, G6PD inhibits ferroptosis by targeting cytochrome P450 reductase, promoting cell growth, metastasis, and tumorigenesis [35]. CBS, a critical enzyme in sulfur amino acid metabolism, plays a role in cysteine synthesis and sulfur metabolism [36]. Apolipoprotein C1 promotes glioblastoma development by inhibiting ferroptosis through reduced CBS activity and increased GSH synthesis [37]. These findings highlight the potential roles of EPAS1, STAT3, G6PD, CYBB, and CBS in asthma, possibly through ferroptosis regulation, and provide new targets for further research. We found that 16HBE cells undergo ferroptosis and inflammation when exposed to IL-13. Notably, IL-13 increases JAK2/STAT3 expression in these cells. STAT3, a key oncogene with dual roles in signal transduction and transcriptional activation, has been identified as a positive regulator of ferroptosis in pancreatic ductal adenocarcinoma (PDAC) cell lines [38]. In breast cancer, STAT3 promotes ferroptosis by increasing ACSL4 expression, while in ulcerative colitis (UC) cell models, STAT3 phosphorylation is downregulated, and the ferroptosis inhibitor Fer-1 can restore its phosphorylation [39]. In high-fat diet-fed mice, STAT3 signaling promotes ferroptosis by upregulating NCOA4-mediated ferritinophagy, contributing to cardiac damage [40]. Our experimental results confirm that STAT3 enhances IL-13-induced inflammation and ferroptosis. The JAK2/STAT3 signaling pathway is involved in numerous BPs [41]. In liver cancer, it induces autophagy [42], while in CRC, the histone deacetylase inhibitor trichostatin A (TSA) reduces JAK2/STAT3 signaling, causing AK2/STAT3, leading to damage in 16HBE cells and contributing to asthma development. EPAS1, a cytokine first cloned in 19G1 phase arrest and apoptosis [43]. Li et al. found that FANCD2 inhibits ferroptosis in osteosarcoma by regulating JAK2/STAT3, thereby promoting osteosarcoma cell viability, migration, invasion, and tumor growth [44]. These findings suggest that IL-13 may exacerbate ferroptosis and inflammation by upregulating J97, contains a bHLH-PAS domain and is primarily expressed in the placenta, heart, lungs, and endothelial cells [45]. It plays a crucial role in cancer, angiogenesis, hematopoiesis, and energy metabolism [46]. Research indicates that EPAS1 induces ferroptosis in clear cell carcinoma by upregulating hypoxia-induced lipid droplet-associated expression [47] and that D-mannose modulates ferroptosis via EPAS1 to alleviate osteoarthritis progression [48]. Our study shows that IL-13 stimulates EPAS1 expression in 16HBE cells. Bioinformatics analysis and experimental validation further confirm that STAT3 positively regulates EPAS1 expression. Additional experiments demonstrated that STAT3 enhances IL-13-induced ferroptosis and inflammation in 16HBE cells by upregulating EPAS1. In summary, IL-13 may promote ferroptosis and inflammation by activating JAK2/STAT3 and EPAS1, leading to 16HBE cell damage and potentially contributing to asthma progression. Several limitations of this study should be acknowledged [49]. First, our experiments were conducted exclusively in the 16HBE cell line, limiting the generalizability of the findings to other cell types. Second, while GPX4 was examined as a key ferroptosis marker, other crucial ferroptosis-related genes, such as SLC7A11 and ACSL4, were not analyzed, which may limit the understanding of ferroptosis mechanisms. Third, the specific phosphorylation sites of JAK2 and STAT3 and their precise effects on EPAS1 expression, inflammation, and ferroptosis were not investigated. Additionally, we did not perform functional validation to determine whether inhibiting JAK2, STAT3, and p-STAT3 could fully reverse IL-13-induced EPAS1 upregulation, inflammation, and ferroptosis. Finally, we did not assess lipid-ROS levels or GSH/GSSG ratios, both of which are critical for evaluating oxidative stress during ferroptosis. Addressing these aspects in future studies will improve the robustness and applicability of our findings.

## Conclusion

This study highlights the critical role of the JAK2/STAT3-EPAS axis in asthma through a combination of bioinformatics analysis and experimental validation. Bioinformatics identified STAT3 and EPAS as key genes closely linked to ferroptosis, with their expression significantly upregulated in asthma and positively correlated with each other. Functional experiments further demonstrated that the JAK2/STAT3 pathway promotes IL-13-induced ferroptosis and inflammation in 16HBE cells by upregulating EPAS1 expression. These findings provide new insights into the molecular mechanisms of asthma and suggest potential therapeutic targets by modulating the JAK2/STAT3-EPAS axis and ferroptosis regulation.

## Supplemental data

**Table S1:** Primers used for qRT-PCR

**Gene**	**Forward primer (5′-3′)**	**Reverse primer (5′-3′)**
JAK2	TGAGTTCGAAGCTAGCAGGGC	ACAGTTGTCTCCACCCTCTCC
STAT3	GGAGAAACAGGATGGCCCAA	ATCCAAGGGGCCAGAAACTG
EPAS1	ATGCTGTCTCTCTTGGCACC	GGTAAGAACCGACAGTGGCA
GPX4	AGTGAGGCAAGACCGAAGTA	GCTTCCCGAACTGGTTACAC
β-actin	CCCTGGAGAAGAGCTACGAG	GGAAGGAAGGCTGGAAGAGT

**Table S2:** Primary antibodies used for western blot

**Gene**	**Manufacturer**	**Product number**	**Theoretical molecular weight**
P-STAT3	Affinity	AF3293	88kDa
STAT3	Affinity	AF6294	86kDa
JAK2	Affinity	AF6022	131KDa
EPAS1	Affinity	DG2928	96KDa
GPX4	Affinity	DF6701	22KDa
β-actin	Zs-BIO	TA-09	42kDa

## Data Availability

All the data are available upon reasonable request to the correspondence author.
